# An fNIRS Investigation of Discrete and Continuous Cognitive Demands During Dual-Task Walking in Young Adults

**DOI:** 10.3389/fnhum.2021.711054

**Published:** 2021-11-16

**Authors:** Tabassum Tahmina Rahman, Nadia Polskaia, Gabrielle St-Amant, Talia Salzman, Diana Tobón Vallejo, Yves Lajoie, Sarah Anne Fraser

**Affiliations:** ^1^Interdisciplinary School of Health Sciences, Faculty of Health Science, University of Ottawa, Ottawa, ON, Canada; ^2^School of Human Kinetics, Faculty of Health Science, University of Ottawa, Ottawa, ON, Canada; ^3^Electronics and Telecommunications Engineering Department, Universidad de Medellín, Medellín, Colombia

**Keywords:** cognitive demand, continuous cognitive task, discrete cognitive task, dual task, fNIRS (functional near infrared spectroscopy), prefrontal cortex (PFC), overground walking, younger adults

## Abstract

**Introduction**: Dual-task studies have demonstrated that walking is attention-demanding for younger adults. However, numerous studies have attributed this to task type rather than the amount of required to accomplish the task. This study examined four tasks: two discrete (i.e., short intervals of attention) and two continuous (i.e., sustained attention) to determine whether greater attentional demands result in greater dual-task costs due to an overloaded processing capacity.

**Methods**: Nineteen young adults (21.5 ± 3.6 years, 13 females) completed simple reaction time (SRT) and go/no-go (GNG) discrete cognitive tasks and n-back (NBK) and double number sequence (DNS) continuous cognitive tasks with or without self-paced walking. Prefrontal cerebral hemodynamics were measured using functional near-infrared spectroscopy (fNIRS) and performance was measured using response time, accuracy, and gait speed.

**Results**: Repeated measures ANOVAs revealed decreased accuracy with increasing cognitive demands (*p* = 0.001) and increased dual-task accuracy costs (*p* < 0.001). Response times were faster during the single compared to dual-tasks during the SRT (*p* = 0.005) and NBK (*p* = 0.004). DNS gait speed was also slower in the dual compared to single task (*p* < 0.001). Neural findings revealed marginally significant interactions between dual-task walking and walking alone in the DNS (*p* = 0.06) and dual -task walking compared to the NBK cognitive task alone (*p* = 0.05).

**Conclusion**: Neural findings suggest a trend towards increased PFC activation during continuous tasks. Cognitive and motor measures revealed worse performance during the discrete compared to continuous tasks. Future studies should consider examining different attentional demands of motor tasks.

## Introduction

Walking was believed to be a relatively simple, automatic motor activity for younger adults (Schneider and Shiffrin, 1977). However, extensive dual-task research has identified that attentional demands play a significant role in the control of walking (Woollacott and Shumway-Cook, 2002; Yogev-Seligmann et al., 2008). Theoretical and experimental works have demonstrated that cognitive and motor domains share neural resources (Miyai et al., 2001; Bayot et al., 2018). Thus, performing cognitive and motor tasks simultaneously (i.e., dual-tasks) may cause interference and performance decrements on one or both tasks (Woollacott and Shumway-Cook, 2002; Leone et al., 2017). As outlined in the capacity sharing theory, once the limit of attentional capacity is exceeded, performance on one or both tasks may decline (Kahneman, 1973; Tombu and Jolicæur, 2003). This makes the effective allocation of attention essential for processing multiple tasks simultaneously. These processes can be measured in the prefrontal cortex (PFC). Greater PFC activation has been associated with increased dual-task attentional demands (Mirelman et al., 2014; Beurskens et al., 2016; Fraser et al., 2016). Therefore, examining neural activity during dual-task walking may provide greater insight into the processing strategies involved in cognitive-motor interference.

Neural activation during walking may be controlled by automatic or executive control processes that utilize two distinct neural networks: direct and indirect locomotor control pathways (Herold et al., 2017). The direct pathway is said to be involved during tasks that require minimal conscious attention whereas interference derived from another task may implicate the indirect pathway, which includes the PFC, to mitigate the added attentional demands (Clark, 2015). The constrained action hypothesis posits that an external focus may divert attention away from body movements and promote greater automatic control (Wulf et al., 2001). However, neuroimaging studies have demonstrated greater PFC involvement during dual-task walking compared to normal walking, which may indicate an interplay between automatic to executive control processes under different attentional demands (Mirelman et al., 2014; Beurskens et al., 2016; Fraser et al., 2016).

Another dual-task performance evaluation method is to measure changes in behavior or the costs of performing a single vs. dual-task. More specifically, manipulating dual-task demands may reveal differences in cognitive (i.e., response time and accuracy) and motor (i.e., gait speed) performance that are associated with changes in PFC activation. Studies have reported mixed findings in the interactions between cognitive and motor domains. For example, a recent review identified that different variations of the serial subtraction task have all led to dual-task gait speed declines due to cognitive-motor interference (Hill et al., 2013; Lu et al., 2015; Pelicioni et al., 2019). However, gait speed changes were not observed during an inhibition task highlighting the importance of examining different sources of interference (Beurskens et al., 2016).

The cognitive performance also proves to be variable across different task types. Participants performing serial subtractions (Mirelman et al., 2014) and choice reaction time (Rosso et al., 2017) tasks demonstrated equal response accuracy between single and dual-tasks. In contrast, single task response accuracy during a working memory task was marginally greater than dual-task accuracy (Fraser et al., 2016). Studies that have directly compared different cognitive tasks have shed light on the differential effects of task type on task performance (Al-Yahya et al., 2011; Pelicioni et al., 2019). For example, walking while concurrently performing a visuomotor reaction time task led to greater motor costs whereas a Stroop task led to greater cognitive costs (Patel et al., 2014). The authors attributed these findings to the type of task whose demands delineated how the task was performed and prioritized within the available processing capacity.

The connotation of “task type” suggests that every task is inherently different. However, the source of cognitive-motor interference is often based on the level of attentional demand (Woollacott and Shumway-Cook, 2002; Patel et al., 2014; Beurskens et al., 2016). Categorizing tasks into discrete and continuous attentional demands, or the degree of expected interference, may, therefore, help to generalize findings between studies. Discrete tasks have a defined end allowing attention to deviate once a response to a stimulus is given (Schmidt and Lee, 2011). In contrast, continuous tasks require more sustained attention as the response to an earlier stimulus may affect a subsequent response (Schmidt and Lee, 2011; Lajoie et al., 2016). These differences become apparent when comparing a working memory task which requires continuous information updating to a reaction time task, which requires less complex processing steps (Bayot et al., 2018). Studies investigating standing balance have demonstrated that continuous tasks contribute to greater automatic postural control (Polskaia et al., 2015; Lajoie et al., 2016). However, this distinction has been scarcely examined during walking tasks.

Functional near-infrared spectroscopy (fNIRS) has become the primary tool for acquiring neurophysiological evidence associated with dual-task costs (Pinti et al., 2020). fNIRS provides a portable method to record, visualize and measure cortical activation during dynamic movements in ways that other neuroimaging technologies (e.g., fMRI) cannot (Menant et al., 2020). By emitting near-infrared light into the cerebral cortex at two wavelengths, fNIRS can detect hemodynamic response changes based on the attenuation properties of oxyhemoglobin (HbO_2_) and deoxyhemoglobin (HbR; Quaresima and Ferrari, 2019). The relative oxygenation changes (ΔHbO_2_ and ΔHbR) can then be inferred using the modified Beer-Lambert law (Kocsis et al., 2006). Previous studies have demonstrated greater PFC activation during continuous cognitive tasks such as working memory and serial seven subtractions while walking (Mirelman et al., 2014; Fraser et al., 2016). Conversely, frontal brain activity did not change between single and dual-task walking when performing an inhibition task (Beurskens et al., 2016). Studies comparing younger and older adults have also revealed that younger adults exhibit unilateral or more localized PFC activation during certain tasks (Cabeza et al., 2002). For example, younger adults activate task-specific regions of the brain such as the dorsolateral prefrontal cortex (dlPFC) during working memory tasks. More specifically, brain activation in the left PFC has been associated with verbal working memory tasks compared to right hemispheric activation during spatial working memory indicating the sensitivity of brain activation to different types of tasks (Reuter-Lorenz et al., 2000).

This study investigated the effects of discrete and continuous attentional demands on dual-task walking. Previous studies have reported inconsistent findings with respect to dual-task neural and performance measures (Pelicioni et al., 2019) and whether or not they differ based on attentional demands. Typically, attentional demand is manipulated within one task type to assess capacity demand limits [i.e., having participants complete serial subtractions by three (easy) or serial subtractions by seven (hard); or in our own work, comparing 1-back performance to 2-back performance (Fraser et al., 2016)]. The current study is novel in that there are four different cognitive tasks paired with walking to examine discrete and continuous attentional demands within the same study. This study uses fNIRS to examine PFC activation as participants complete two discrete and two continuous cognitive tasks that were designed to evaluate processing speed, response inhibition, and working memory. It is hypothesized that increased PFC activation and poorer performance (i.e., slower walk speed, longer response times, and lower accuracy rates) will be observed during the dual compared to single task conditions. Similarly, continuous cognitive demands, which involve sustained attention, are expected to produce greater PFC activation changes and greater decrements in performance compared to discrete tasks.

## Materials and Methods

### Participants

Nineteen right-handed young adults (21.5 ± 3.6 years, 13 females) between the ages of 18 and 35 years were recruited *via* social media and flyers to participate in this study. All participants completed a health phone screening to ensure that they met the study’s inclusion criteria: right-handedness, ability to walk 15 meters without assistance, free of self-reported hearing impairments, or neuromuscular complaints that may compromise their walking performance. A brief overview of participant demographics is provided in Table 1. This study was approved by the University of Ottawa Research Ethics Board and participants provided written informed consent prior to participation.

**Table 1:** Mean (*M*) and standard deviation (*SD*) for the participant demographics and health questionnaires.

Measure	*M*	*SD*
Mean Age (years)	21.6	3.6
Females	13	
Males	6	
Education (years)	13.5	1.80
Self-paced walking (m/s)	0.84	0.16
Beck Depression Inventory	2.3	2.80
Falls Efficacy Scale International	33.8	12.3
Short Physical Performance Battery	11.6	0.65

*A Beck Depression Inventory (/63) score of less than four indicates a healthy mental state. The Short Physical Performance test (/12) is used to identify the physical state in which higher values indicate better physical performance. Furthermore, the Falls Efficacy Scale (range: 16–64 points) is used to identify participants with a fear of falling such that higher values indicate greater concerns*.

### Apparatus

**Figure 1:**
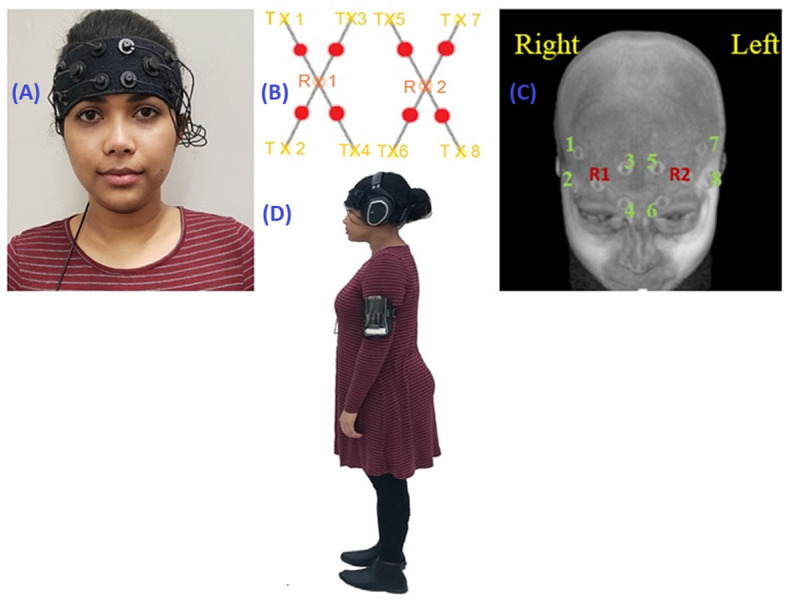
**(A)** Participant wearing the fNIRS device. **(B)** fNIRS optode configuration. **(C)** fNIRS optode configuration mapped onto an MRI scan. Rx1 and Rx2 are detectors; Tx1 through Tx8 are channels. **(D)** Fully instrumented participant with fNIRS, headphones, and voice recorder.

For the duration of the experiment, participants wore a voice recorder on their upper arm (Philips), headphones (Sennheiser RS 165), and personal walking shoes. Figure 1 illustrates a fully instrumented participant. Experimenters presented the task instructions over a microphone (Audix) which could be heard in the participants’ headphones along with the start and stop cues and the cognitive task stimuli. A continuous wave, non-invasive OctaMon fNIRS system (Artinis Medical Systems, The Netherlands) was used to record ΔHbO_2_ and ΔHbR. It consisted of eight channels covering the PFC (Figure 1).

### Experimental Tasks

#### Cognitive Tasks

Each cognitive task was programmed in E-Prime software (Psychology Software Tools, Pittsburgh, PA). The cognitive tasks described below have been used in published dual-task studies (Fraser et al., 2016; Lajoie et al., 2016; St-Amant et al., 2020; Salzman et al., 2021). Moreover, the discrete tasks were made up of a simple reaction time (SRT) and go/no-go inhibition task (GNG) whereas the continuous tasks were composed of n-back (NBK) and double number sequence (DNS) working memory tasks. The single cognitive (SC) condition had participants stand with their feed shoulder-width apart as they performed each cognitive task. In addition, response times and accuracy rates were recorded for each task. Vocal response times (ms) were derived in Audacity (v. 2.3.1) by measuring the time from the onset of the stimulus to the onset of the participant’s response. Average response times were calculated for correct responses only. The accuracy rate (%) was calculated based on the number of correct responses given by the participants out of the total possible correct responses.

SRT: Participants listened to a random sequence of beeps (2,850 Hz at 99 dB) and responded by saying the word “top” as fast as possible following each beep. Errors were tabulated following each stimulus when the participant did not respond “top” before the next stimulus was presented. A response time was calculated based on the time between the stimulus onset and the onset of the participant’s response.

GNG: Participants listened to high- (2,850 Hz at 99 dB) and low-pitched (970 Hz at 95 dB) beeps. They were then instructed to respond “top” as fast as possible to the high-pitched beeps while inhibiting their responses to the low-pitched ones. Errors were noted if participants missed a high beep or if they responded to a low beep. A response time was calculated based on the time between the onset of the high beep and the participant’s response.

NBK: Participants listened to a continuous series of single-digit numbers that were presented every 2.5 s and were instructed to respond with the number they heard two numbers back. The numbers were pseudo-randomly presented to ensure that there were no repeats (e.g., 1-1) or ordered series (e.g., 1-2-3). Errors were registered if participants did not respond with the correct number or did not respond before the next number was presented. A response time was calculated based on the time between the presentation of the number and the participant’s response.

DNS: Three-digit numbers were presented every 2 s and participants had to silently count the number of times they heard two pre-designated digits within the sequence. At the end of the block, participants were asked to provide the totals for each number they tracked. This cognitive task differs from the other tasks in that participants only responded at the end of the block. As a result, a response time was not derived for this task. Errors were calculated from the difference between the total possible correct responses and the participants’ tallies of the individual target digits.

#### Motor Tasks

In the single motor (SM) condition, participants were asked to walk at their self-selected pace with instructions to “walk to a meeting that you are not late for.” Participants then walked along a 10-meter (m) pathway which was marked on either end with a horizontal line. At the end of the block, participants stopped walking and stood in a place so that the experimenter could measure the distance they walked over the course of the block. Gait speed could then be calculated by dividing the distance the participant walked with the fixed duration of the block.

#### Dual-Tasks

In the dual-task (DT) condition, participants walked at their self-selected pace while simultaneously performing each of the cognitive tasks. Participants were instructed to pay equal attention to both the motor and cognitive tasks, as described in previous studies (Laguë-Beauvais et al., 2015; Fraser et al., 2016). Response time, accuracy, and gait speed were measured following the same guidelines presented in the SC and SM conditions.

### Experimental Protocol

**Figure 2:**
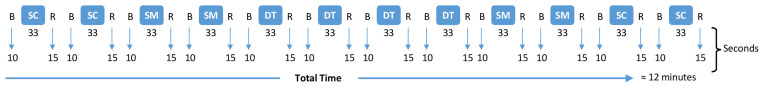
Experimental protocol for a run. B = baseline (fNIRS quiet standing); R = rest; SC = single cognitive (SRT, GNG, NBK or DNS in a randomized run order while standing); SM = single motor (usual walking); DT = dual-task (cognitive task and walking).

The experimental protocol consisted of four runs, one for each cognitive task, that were randomized across participants. Figure 2 provides an overview of the 12 blocks that made up one run: four SC, four SM, and four DT. Participants were given a familiarization phase for each condition and cognitive task to ensure that they understood the instructions prior to performing the recorded run. Participants needed to achieve an accuracy >70% on each respective cognitive task during familiarization in order to proceed to the experimental blocks.

The order of the blocks was counterbalanced to control for learning (Leff et al., 2011) and practice effects (Allen, 2017). A 10 s baseline was collected before beginning each block and was followed by the 33 s task and a 15 s rest period (Menant et al., 2020). The rest period ensured that enough time had passed for the hemodynamic response to revert to the baseline before the next block was to begin (Herold et al., 2017). Similarly, the 33 s block duration ensures a sufficient measurement time to capture hemodynamic response changes (Menant et al., 2020).

After the experiment, participants completed neuropsychological and physical assessments including the shortened Beck Depression Inventory (Beck et al., 1996), the Short Physical Performance Test (SPPB; Guralnik et al., 1994), as well as the Falls Efficacy Scale International (Delbaere et al., 2010). These tests were used to assess baseline cognitive and physical characteristics of the younger adult sample and are presented in Table 1.

### fNIRS Acquisition

**Figure 3:**
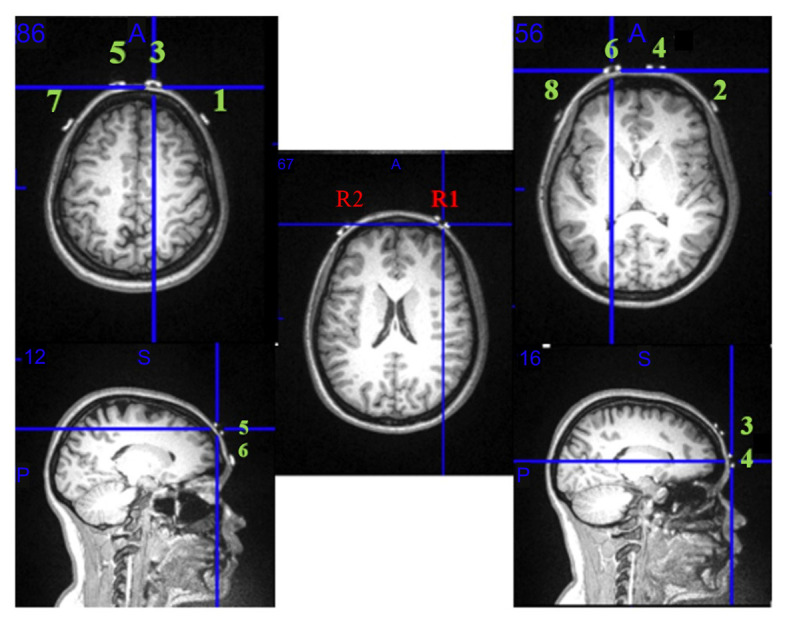
fNIRS optode configuration mapped onto an MRI scan. MRI demonstrates that all the optodes and receivers on the fNIRS are positioned over the right and left Brodmann area 10. R1 and R2 represent detectors; the green numbers 1 through 8 are the fNIRS infrared light emitters.

Head circumference was measured for each participant to facilitate fNIRS device placement on FP1/FP2 using the modified international EEG 10/20 system (Okamoto et al., 2004). FP1 and FP2 were aligned within 10% of the distance from the nasion to the inion on the midsagittal plane while the position of electrode Cz was fixed at the vertex of the head. The goal was to cover the entire PFC including the dorsolateral, ventrolateral, and medial regions. One participant was scanned in an MRI scanner, with fiducial markers placed at each of the eight fNIRS optode locations to support that these regions were being captured using the fNIRS device (Figure 3). Prior to beginning acquisitions, the fNIRS signal was visualized online to ensure that there was a strong signal (e.g., with the heartbeat) as indicated by a synchronous waveform and without any noise from ambient light contamination (Kirilina et al., 2013). In case the fNIRS device shifted during walking, references were marked with a highlighter on the nasion as well as left/right pre-auricular points to fix the anatomical points.

fNIRS data was collected *via* Bluetooth and sampled at a frequency of 10 Hz. Two wavelengths (690 and 830 nm) were used to measure ΔHbO_2_/ΔHbR and were visualized in Oxysoft (v. 3.0.97.1). The raw data was then converted to concentrations using the Modified Beer-Lambert law (Villringer and Chance, 1997) and the differential pathlength factor was set according to the expected pathlength for the participant’s age (Scholkmann and Wolf, 2013). The signal was also visually inspected to ensure that there were no abrupt spikes due to motion artifacts. A wavelet analysis was conducted to modify the frequency parameters in the bandpass filter (0.5 and 0.01) and to account for physiological artifacts including respiration changes due to vocalization (Hocke et al., 2018; Pinti et al., 2019). The neural data were processed offline using a custom MATLAB script (v. R2018a). The script eliminated motion artifacts by removing outliers that were 2.5 SD from the mean. An average ΔHbO_2_ and ΔHbR value was then calculated in μM for each task (SC, SM, DT), each difficulty level (SRT, GNG, NBK, DNS), and each Hemisphere (Left and Right) from the changes in signal between the baseline and active conditions.

### Statistical Analyses

The behavioral and neural data were analyzed with SPSS IBM (v. 23). For all ANOVA comparisons, statistical significance was set at *α* = 0.05. All *post hoc* analyses were Bonferroni corrected and a Greenhouse-Geisser *p-*value was reported if Mauchly’s Test of Sphericity was violated.

#### Behavioral Data

Behavioral data analyses included accuracy (%), gait speed (m/s), and vocal response time (ms). Each measure was checked for outliers that were 2.5 standard deviations from the group mean. No outliers were found using this method.

*Accuracy (%)*: Mean accuracy rates were calculated for each cognitive task during SC and DT conditions. A 2 × 4 repeated measures ANOVA compared conditions (SC/DT) and the four cognitive tasks (SRT, GNG, NBK, DNS).

*Response time (ms)*: Mean response times were measured for SRT, GNG, and NBK tasks for both SC and DT conditions. Note, a response time was not calculated for the DNS task since participants only responded at the end of the block. A 2 × 3 repeated measures ANOVA was calculated across two conditions (SC/DT), two discrete and one continuous cognitive task (SRT, GNG, NBK).

*Gait (m/s)*: Mean gait speed was calculated across a 2 × 4 repeated measures ANOVA that compared conditions (SM/DT) and the four cognitive tasks (SRT, GNG, NBK, DNS).

#### Neural Data

Mean ΔHbO_2_ and ΔHbR hemisphere data (left PFC and right PFC) were analyzed using paired sample t-tests to test for hemispheric differences in SC and SM for each cognitive task (SRT, GNG, NBK, DNS) separately. There were hemispheric differences in ΔHbO_2_ SM SRT (*p* = 0.005) and SC NBK (*p* = 0.045). There were no significant hemispheric differences in ΔHbR (*p-values* >0.057). Since the t-tests revealed differences between the left and right hemisphere, 2 × 2 × 4 repeated measures ANOVAs were conducted to compare ΔHbO_2_ and ΔHbR across condition (SC/SM, DT), hemisphere (left, right), and cognitive tasks (SRT, GNG, NBK, DNS). This statistical approach allows us to assess if there are discrete vs. continuous task differences, if there are dual vs. single task differences (i.e., SM vs. DT or SC vs. DT), any hemispheric differences, and if there are any interactions with these factors. In addition, we have applied this approach to a completed study with the exact same design, tasks, and protocols but was conducted with older adults (Salzman et al., 2021).

## Results

### Behavioral Findings

#### Cognitive Response Accuracy

**Figure 4:**
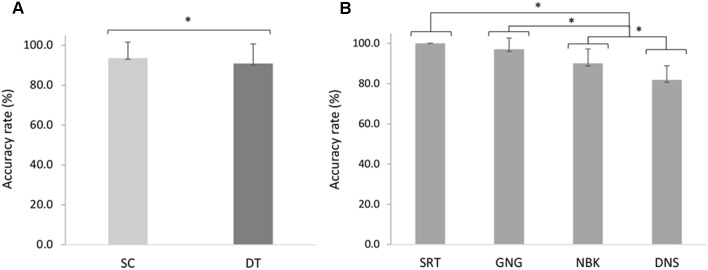
Mean changes in accuracy rates (%) across **(A)** single (SC) and dual (DT) tasks (*F*_(1, 18)_ = 15.42, *p* = 0.001, $\eta_{\text{p}}^{2}$ = 0.46). **(B)** Simple reaction time (SRT), go/no-go (GNG), n-back (NBK) and double number sequence (DNS) accuracy rates across cognitive demands (*F*_(3, 54)_ = 63.82, *p* < 0.001, $\eta_{\text{p}}^{2}$ = 0.78). Error bars represent standard deviation and (*) denotes a significant difference (*p* < 0.05).

The 2 × 4 repeated measures ANOVA indicated a significant main effect of task *F*_(1, 18)_ = 15.42, *p* = 0.001, $\eta_{\text{p}}^{2}$ = 0.46 whereby *post hoc* analyses revealed decreased accuracy in DT (*M* = 90.8%, *SD* = 9.82%) compared to SC (*M* = 93.7%, *SD* = 7.99%; *p* < 0.001). Participants also exhibited a main effect of demand *F*_(3, 54)_ = 63.82, *p* < 0.001, $\eta_{\text{p}}^{2}$ = 0.78, with decreasing accuracy across the SRT (*M* = 100%, *SD* = 0.0%), GNG (*M* = 97.1%, *SD* = 5.61%), NBK (*M* = 90.0%, *SD* = 7.15%), and DNS (*M* = 81.9%, *SD* = 6.95%). *Post hoc* analyses revealed that the SRT was more accurate than the NBK (*p* < 0.001) and DNS (*p* < 0.001). The GNG was also more accurate than NBK (*p* = 0.01) and DNS (*p* < 0.001), while NBK was more accurate than the DNS (Figure 4).

#### Response Time

**Figure 5:**
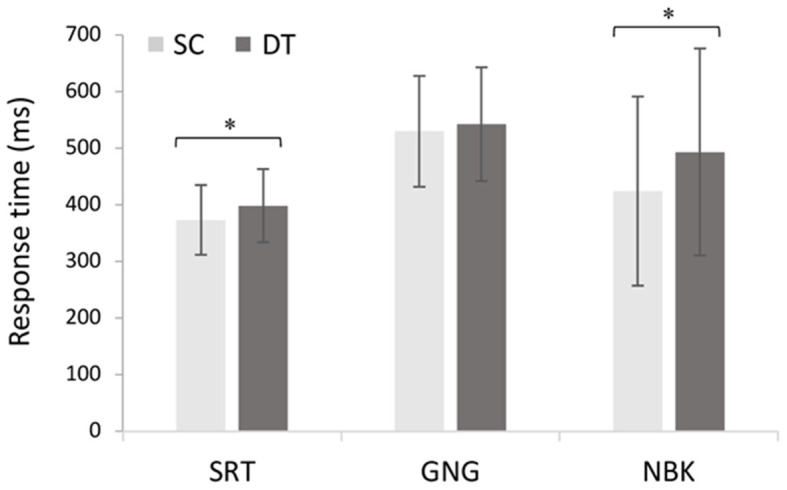
Mean changes in response time across single cognitive (SC) and dual-tasks (DT) with increasing cognitive demand (*N* = 19, *F*_(2, 36)_ = 4.1, *p* = 0.026, $\eta_{\text{p}}^{2}$ = 0.18). Error bars represent standard deviation and (*) denotes a significant difference (*p* < 0.05).

The 2 × 3 repeated measures ANOVA comparing single and dual-tasks with the cognitive demands (SRT, GNG, NBK) revealed a main effect of task *F*_(1, 18)_ = 20.65, *p* < 0.001, $\eta_{\text{p}}^{2}$ = 0.53 with *post hoc* analyses revealing slower response times in DT (*M* = 481 ms, *SD* = 137 ms) than SC (*M =* 445 ms, *SD* = 133 ms; *p* < 0.001). A main effect of demand *F*_(2, 36)_ = 10.61, *p* < 0.001, $\eta_{\text{p}}^{2}$ = 0.37 also revealed longer response times during both GNG (*M* = 536 ms, *SD* = 98.0; *p* < 0.001) and NBK (*M* = 459 ms, *SD* = 176 ms; *p* = 0.03) than SRT (*M* = 386 ms, *SD* = 63.3 ms). These results were superseded by a significant task × demand interaction *F*_(2, 36)_ = 4.1, *p* = 0.026, $\eta_{\text{p}}^{2}$ = 0.18 where *post hoc* analyses revealed that DT response times (*M*_SRT DT_ = 398 ms, *SD* = 64.4 ms, *p* = 0.005; *M*_NBK DT_ = 503 ms, *SD* = 182.4 ms, *p* = 0.004) were significantly slower than SC for the SRT and NBK tasks (*M*_SRT SC_ = 373 ms, *SD* = 61.3 ms; *M*_NBK SC_ = 423 ms, *SD* = 167 ms; Figure 5).

#### Gait Speed

**Figure 6:**
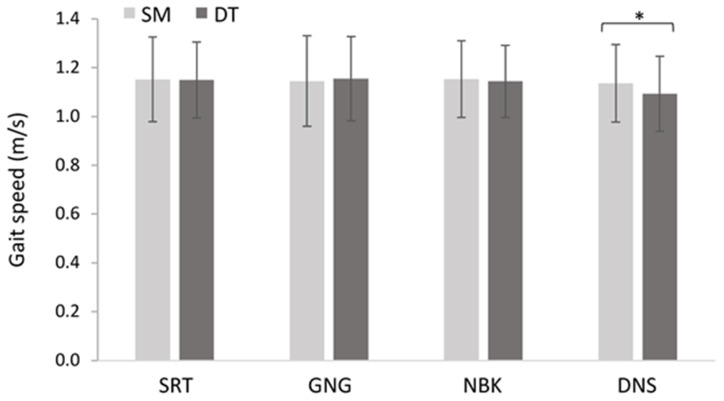
Mean changes in gait speed across single motor (SM) and dual-task (DT) blocks (*N* = 19, *F*_(3, 54)_ = 5.579, *p* = 0.002, $\eta_{\text{p}}^{2}$ = 0.24). Error bars represent standard deviation and (*) denotes a significant difference (*p* < 0.05).

The 2 × 4 repeated measures ANOVA between single/dual-tasks and the cognitive demands (SRT, GNG, NBK, DNS) revealed a main effect of task *F*_(1, 18)_ = 6.18, *p* = 0.02, $\eta_{\text{p}}^{2}$ = 0.26. *Post hoc* analyses revealed that participants walked slower during DT (*M* = 1.13 m/s, *SD* = 0.17 m/s) compared to SM (*M* = 1.15 m/s, *SD* = 0.16 m/s). These results were superseded by a significant task × demand interaction *F*_(3, 54)_ = 10.2, *p* < 0.001, $\eta_{\text{p}}^{2}$ = 0.36. *Post hoc* analyses revealed that DT gait speed (*M* = 1.09 m/s, *SD =* 0.15 m/s) was significantly slower than SM (*M* = 1.14 m/s, *SD* = 0.15 m/s) during the DNS task (*p* < 0.001; Figure 6).

### Cerebral Hemodynamics

#### Hemodynamic Response During Single Motor and Dual-Task

**Figure 7:**
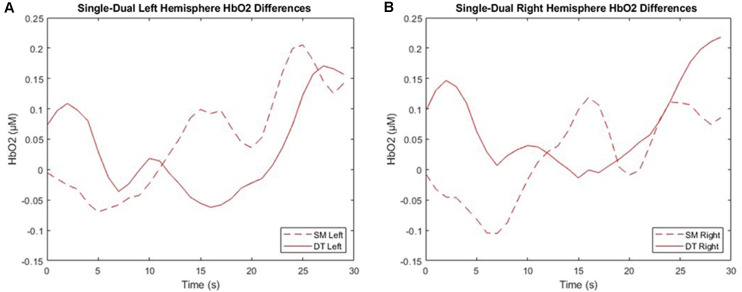
Left **(A)** and right **(B)** mean hemispheric changes in cerebral oxygenation (ΔHbO_2_) between single motor (SM) and dual-task (DT) blocks. The right hemisphere consists of channels 1–4 and the left hemisphere consists of channels 5–8. This data is the group average post-processed with baseline subtraction, filtering, and outlier correction.

2 × 2 × 4 repeated measures ANOVAs were used to examine the ΔHbO_2_ and ΔHbR interactions across task (SM, DT), hemisphere (left, right), and cognitive demand (SRT, GNG, NBK, DNS). Findings revealed a three-way ΔHbO_2_ interaction *F*_(3, 54)_ = 5.73, *p* = 0.002, $\eta_{\text{p}}^{2}$ = 0.24 where *post hoc* tests revealed a trend towards significance in ΔHbO_2_ (*p =* 0.06) in which DT (*M* = −0.001 μM, *SD* = 0.17 μM) was greater than SM (*M* = −0.024 μM, *SD* = 0.10 μM) in the right hemisphere during the DNS cognitive task. ΔHbR findings indicated a significant interaction between task, hemisphere, and demand *F*_(3, 54)_ = 5.579, *p* = 0.002, $\eta_{\text{p}}^{2}$ = 0.24. The *post hoc* analyses revealed that during DNS in the right hemisphere, DT (*M* = 0.004 μM, *SD* = 0.05 μM) was greater than SM (*M* = −0.113 μM, *SD* = 0.23 μM) but this interaction only trended towards significance following the Bonferroni correction (*p-value* > 0.06; Figure 7).

#### Hemodynamic Response During Single Cognitive and Dual-Task

2 × 2 × 4 repeated measures ANOVAs were used to compare ΔHbO_2_ and ΔHbR interactions between task (SC, DT), hemisphere (left, right) and demand (SRT, GNG, NBK, DNS). Findings revealed a three-way interaction between task, hemisphere, and demand for ΔHbO_2_ *F*_(3, 54)_ = 4.08, *p* = 0.01, $\eta_{\text{p}}^{2}$ = 0.19. *Post hoc* analyses revealed a marginally significant interaction (*p =* 0.05) during the SC NBK task such that ΔHbO_2_ in the right hemisphere (*M* = 0.69 μM, *SD* = 0.27 μM) was greater than the left (*M* = 0.003 μM, *SD* = 0.30 μM). ΔHbR findings indicated a significant interaction between task, hemisphere, and demand *F*_(3, 54)_ = 4.635, *p* = 0.006, $\eta_{\text{p}}^{2}$ = 0.21 but this interaction was not significant following *post hoc* analyses (*p-values* > 0.21).

## Discussion

The aim of the current study was to compare the effects of discrete and continuous cognitive demands on dual-task walking in young adults. More specifically, this study investigated neural, cognitive, and motor performance costs as participants walked with cognitive tasks that required either short intervals or sustained attention. Continuous cognitive tasks were expected to be associated with the greatest PFC activation changes (i.e., ΔHbO_2_, ΔHbR) as well as diminished behavioral performance (i.e., response time, accuracy, and gait speed) in comparison to discrete tasks. Findings partially supported this hypothesis in that performance declines were mainly observed during the continuous tasks (i.e., NBK and DNS) and performance decreased during dual compared to single tasks. Interactions between the cognitive tasks along with single and dual-task conditions revealed worse dual-task performance during the continuous tasks. However, differences in PFC activity were not observed at each level of cognitive demand. Secondly, dual-tasks were expected to elicit greater PFC activation and performance decrements compared to single tasks. PFC findings pointed towards an increase in the DT compared to the SM condition and greater activation during the NBK continuous task in the right hemisphere during the DT compared to the SC condition.

### Cognitive Response Time and Accuracy

Cognitive performance findings revealed the main effects of accuracy between SC and DT conditions along with continuous and discrete cognitive demands. As expected, the dual-tasks elicited less accurate responses than the single tasks across each cognitive demand level. The continuous tasks were also less accurate than the discrete tasks; such that the DNS was the least accurate followed by the NBK, GNG, and SRT, respectively. These findings are in line with the attentional demand differences between discrete and continuous tasks (Schmidt and Lee, 2011; Lajoie et al., 2016). For example, the DNS task involved mental arithmetic as participants tallied the target digits and working memory to actively adapt their responses to the incoming stimuli. In contrast, participants did not have to be as attentive to the SRT task since each stimulus and response was identical across the block. Thus, the complexity and number of processing steps in a continuous vs. discrete cognitive task support the decrease in accuracy performance. Similar findings were obtained in studies that increased the retention difficulty and amount of information manipulation that was required to complete a cognitive task (Hill et al., 2013; Fraser et al., 2016).

Response time findings also indicated a decrease in performance between single and dual-tasks. This is in line with the capacity sharing theory; attending to one task leaves limited cognitive resources to perform a secondary task (Kahneman, 1973). However, deciding on how this capacity is allocated may depend on the task at hand. In this study, only the SRT and NBK tasks elicited slower response times between single and dual-tasks. Differences were expected during the NBK since it required more sustained attention and a larger proportion of processing resources than discrete tasks. In contrast, the discrete SRT task indicated that dividing attention while walking, even at intermittent intervals, may disrupt cognitive performance (Rosso et al., 2017).

Differences between single and dual-tasks were not observed during the GNG task, but overall, response times were significantly slower than the SRT. This is consistent with a previous study that revealed slower GNG response times than the SRT during a standing balance task (Lajoie et al., 2016). In the present study, slowing response times may have been an adaptive strategy to mitigate the demands of GNG compared to SRT. Additionally, participants maintained a high level of response accuracy despite slower response times in the discrete tasks. However, this was not observed during the continuous NBK task in which response times decreased but did not significantly benefit accuracy. This demonstrates that even across cognitive performance measures, lower attentional demands may ultimately result in lower overall dual-task costs.

### Gait Speed

Motor performance findings partially support the initial hypothesis in that gait speed decrements between single and dual-tasks were only observed during the DNS continuous task. The DNS is attention-demanding because it involves working memory and mental tracking as participants hold and manipulate the two target digits amidst incoming stimuli (Al-Yahya et al., 2011; Bayot et al., 2018). Gait speed costs have been previously reported in both these domains and to a greater extent during continuous compared to discrete tasks (Hill et al., 2013; Mirelman et al., 2014). This is because dual-task costs increase with the degree of interference between shared cognitive pathways (Al-Yahya et al., 2011). Therefore, findings from this study suggest that there was minimal interference between cognitive and motor control during the discrete tasks. This is supported by reports in which a go/no-go discrete task did not elicit gait speed differences between single and dual-task walking (Beurskens et al., 2016).

Additionally, interference can be minimized by tasks that promote automatic locomotor control which may have occurred during the other cognitive tasks (Wulf et al., 2001). This contradicts previous reports suggesting that the sustained attentional demands of continuous tasks better promote automatic postural control during a standing balance task (Polskaia et al., 2015; Lajoie et al., 2016). This brings into question the effect of motor task difficulty (i.e., walking vs. standing) on cognitive-motor interference (Patel et al., 2014; Lin and Lin, 2016; Maidan et al., 2018). As demonstrated by single and dual-task costs in this study, walking is arguably more attention-demanding than standing. Therefore, continuous demands may better promote automaticity during a standing balance task whereas discrete demands have the same effect but on walking performance.

Based on this interpretation, gait speed decrements would also be expected during the NBK task. However, NBK gait speed was maintained between single and dual-tasks. One reason for this may be auditory-motor entrainment, which is caused by auditory-motor coupling. Previous studies have demonstrated that auditory and motor stimuli with overlapping frequencies can align and enhance gait performance (Thaut, 2015; Moumdjian et al., 2018). This process is commonly used in Parkinson’s gait rehabilitation to create rhythmic auditory cues to train walking performance (Ghai et al., 2018). The combination of consistent responses and equally dispersed stimuli during the NBK task may have primed the motor system to a greater extent than the DNS task, which did not include active responses during the task (Thaut, 2015). In addition, the consistent rhythm of stimuli may optimize temporal gait performance by creating a time limit for the motor system to execute the walking motion before the next stimulus (Thaut, 2015). This is supported by the present study’s findings in that NBK gait speed, and, NBK DT gait speed, was the fastest amongst all the tasks.

### Cerebral Hemodynamics

#### Single Motor Compared to Dual-Task

HbO_2_ findings comparing SM and DT revealed similar levels of PFC activation between single and dual discrete tasks. The lack of neural differences suggests that participants were able to manage the dual-task demands by adapting their behavior rather than increasing PFC recruitment. For example, response times were slower, and accuracy decreased during the dual compared to single tasks. From a motor performance perspective, there were no differences in gait speed during the discrete tasks, which supports greater automatic control or motor prioritization and decreased recruitment of the PFC. These findings are consistent with previous works that demonstrated decreased PFC activity between single and dual-tasks and, therefore, greater automatic control during a go/no-go task while walking (Beurskens et al., 2016).

In contrast to the hypothesis, ΔHbO_2_ differences were not obtained during the NBK continuous task. Interestingly, two studies have identified decreased PFC activity between single and dual-tasks (Beurskens et al., 2014; Lin and Lin, 2016). The first study demonstrated this interaction during an n-back task in which the findings were attributed to neural plasticity and reorganization that occurs in younger adults when faced with complex cognitive demands (Lin and Lin, 2016). The second study demonstrated that decreased PFC activity resulted from obstructed vision during dual-task walking (Beurskens et al., 2014). The tasks in this study were purposely chosen to minimize motor-motor or motor-visual interferences. In line with the first study’s findings, our study demonstrated the younger adults’ adaptability when faced with differing levels of attentional demands.

In comparison, an interaction was obtained between single and dual-task conditions, hemispheres, and cognitive demands but only trended towards significance following *post hoc* analyses. More specifically, ΔHbO_2_ during the DNS dual-task was greater than the single task in the right hemisphere. This is in line with the continuous task gait speed findings in that decreased gait speed may be indicative of a shift from automatic to executive control. This is also supported by previous studies that have demonstrated increased PFC activation between single and dual working memory tasks (Hill et al., 2013; Lu et al., 2015). Also, in line with the capacity sharing model, greater task demands may necessitate neural upregulation in order to perform the task (Tombu and Jolicæur, 2003). However, there is a limit to this upregulation after which the PFC cannot be kept sufficiently activated despite increasing demands.

#### Single Cognitive Compared to Dual-Task

A second comparison between SC and DT trended towards significance between right and left PFC hemispheres such that ΔHbO_2_ was greater in the right hemisphere during the NBK SC task. Spatial n-back tasks have been previously associated with right PFC lateralization (Kane and Engle, 2002). Although the NBK used in this study did not involve the visual localization of stimuli, continuous tracking and the demand of the task may have contributed to greater right hemisphere activation in a similar manner as spatial n-back tasks. Greater activation in the right PFC also aligns with the ventral attentional network, which is activated in response to stimulus-driven attention and working memory processes (Bayot et al., 2018). Characteristic of the NBK task, the right PFC is more active during effortful memory retrieval and monitoring of stimuli (Henson et al., 1999; Cabeza et al., 2003).

## Limitations

The current study has several limitations including the quantification of motor performance exclusively using gait speed. As demonstrated in previous studies, additional gait parameters such as stride variability and cadence may contribute to the understanding of the interplay between automatic executive control processes during discrete and continuous tasks (Lövdén et al., 2008; Beurskens et al., 2016).

Additionally, this study was limited to hemodynamic response measures in the PFC. The PFC is a reliable measure of executive control processes during discrete and continuous tasks (Pelicioni et al., 2019). However, previous studies have demonstrated activation in motor regions that are associated with automatic and executive locomotor control pathways (Miyai et al., 2001; Lu et al., 2015). In addition, this study did not use short channels to filter extracerebral layer contamination from the fNIRS signal. This is because the fNIRS device had fixed optode positions that did not permit short channels. Short channels may only be advantageous if a high quality of data can be obtained from the short channels without producing more noise (Menant et al., 2020).

## Conclusion

A large body of literature has focused on unraveling the neural correlates of the automatic and executive control of walking (Pelicioni et al., 2019). Dual-task studies demonstrated that exceeding the available processing capacity leads to decrements in cognitive and motor performance. However, findings often differ between studies based on the type of task. This study categorized cognitive tasks based on discrete and continuous attentional demands. The discrete demands resulted in less decrements in cognitive and motor performance than the continuous tasks. More specifically, there were minimal dual-task costs during the discrete vs. continuous tasks in line with the capacity sharing theory. Neural findings were only marginally significant in our most demanding continuous task. Younger adults may, therefore, be able to adapt to increased cognitive demands by modifying their cognitive and motor performance rather than increasing PFC recruitment. Future studies should investigate whether this interaction exists during discrete and continuous motor attentional demands.

## Data Availability Statement

The raw data supporting the conclusions of this article will be made available by the authors, without undue reservation.

## Ethics Statement

The studies involving human participants were reviewed and approved by University of Ottawa Research Ethics Board. The patients/participants provided their written informed consent to participate in this study. Written informed consent was obtained from the individual(s) for the publication of any potentially identifiable images or data included in this article

## Author Contributions

TR, NP, GSt-A, YL, and SF all contributed to the development, design, and study protocol. DV developed the script to preprocess the fNIRS data. TR, TS, and SF analyzed the results. All authors contributed to the write-up and editing of the manuscript. All authors contributed to the article and approved the submitted version.

## Conflict of Interest

The authors declare that the research was conducted in the absence of any commercial or financial relationships that could be construed as a potential conflict of interest.

## Publisher’s Note

All claims expressed in this article are solely those of the authors and do not necessarily represent those of their affiliated organizations, or those of the publisher, the editors and the reviewers. Any product that may be evaluated in this article, or claim that may be made by its manufacturer, is not guaranteed or endorsed by the publisher.
